# Real-world efficacy and safety outcomes of acalabrutinib in chronic lymphocytic leukemia: primary results of a French multicentre observational study (NAOS)

**DOI:** 10.1007/s00277-025-06498-5

**Published:** 2025-07-30

**Authors:** Anne Quinquenel, Stéphane Leprêtre, Marie Sarah Dilhuydy, Lucile Bussot, Zora Marjanovic, Omar Benbrahim, Romain Guièze, Alberto Santagostino, Caroline Dartigeas, Pierre Feugier, Damien Roos-Weil, Ghandi Damaj, Kamel Laribi, Loic Ysebaert, Vincent Levy

**Affiliations:** 1 Hôpital Robert Debré, Reims, France; 2 Centre Henri-Becquerel, Rouen, France; 3 Hôpital du Haut-Lévêque, Bordeaux, France; 4 CHU Grenoble Alpes, Grenoble, France; 5 Hôpital Saint-Antoine, Paris, France; 6 CHR Orléans, Orléans, France; 7 CHU Estaing, Clermont-Ferrand, France; 8 Hôpital Simone Veil, Troyes, France; 9 CHU Tours, Tours, France; 10 CHU Régional de Nancy, Vandoeuvre lès Nancy, France; 11 CHU Pitié Salpêtrière, Paris, France; 12 CHU Caen, Caen, France; 13 CH le Mans, Le Mans, France; 14 IUCT-Oncopôle, Toulouse, France; 15 Hôpital Avicenne, Bobigny, France

**Keywords:** Chronic lymphocytic leukemia, Acalabrutinib, Real-world efficacy, Real-world safety, Second-generation BTK inhibitor

## Abstract

Acalabrutinib is a second-generation BTK inhibitor approved for the treatment of chronic lymphocytic leukemia (CLL) after demonstrating efficacy and safety in clinical trials. NAOS, a real-world study, complements these trials with real-world (rw) acalabrutinib data generated from clinical practice. This retrospective, non-interventional, longitudinal study was conducted in 59 sites in France in adult patients initiating acalabrutinib for CLL in 2021–2022.The study assessed rw use (time to discontinuation, TTD) and effectiveness (rw progression free survival, rwPFS) in first line (L1) and relapsed/refractory setting (L2+). At the time of the analysis, data were collected from routine medical records since the treatment start up to the end of 2022. Of the 485 enrolled patients, 58.8% were in L1 and 41.2% in L2+. Median age was 73 (L1) and 77 (L2+) years. Of them, 55.1% had a prior cardiovascular history, 25.1% del(17p)/*TP53*m, and 67.4% were u*IGHV*. Among 188 patients initiated in 2021 (119 L1; 69 L2+), 31.4% discontinued treatment, 14.9% of whom due to adverse event (AE). The 12-month rwPFS rate was 93.1% (88.5–97.7) in L1 and 87% (79.0-94.9) in L2 + patients with no significant difference based on del17p/*TP53*m (*p* = 0.21) and on *IGHV* status (*p* = 0.34). AEs leading to treatment changes occurred in 40 patients (21.3%), grade 3/4 AE in 9.6%, while cardiac disorders occurred in only 2.1% of patients. NAOS, one of the largest real-world cohorts of acalabrutinib-treated CLL patients in Europe, showed effectiveness and safety consistent with clinical trials, despite an older population with more cardiovascular comorbidities.

## Background

Chronic Lymphocytic Leukaemia (CLL) is one of the most prevalent adult leukaemia in Western countries [1]. The incidence is estimated at 4 to 6 per 100 000 people per year in Europe and USA [2–4] and increases dramatically with age to over 30 per 100 000 people per year from the age of 80 years [5]. CLL primarily affects older people, with more than 44% of patients aged 75 years and older [6, 7]. The overall 5-year survival rate for CLL has substantially improved in recent years with current estimates ranging from 87 to 88.5% in the Western countries [1, 8]. Survival rates decrease consistently with age, with 93%, 88% and 75% 5-year survival for patients aged ≤ 65, 66–75 and over 75 years, respectively [8].

The treatment landscape of CLL has changed significantly in recent years with the advent of targeted therapies such as B-cell lymphoma 2 (BCL2) and Bruton’s tyrosine kinase (BTK) inhibitors [1, 2]. Among the latter, acalabrutinib has emerged as a second-generation BTK inhibitor, offering improved selectivity and reduced off-target kinase inhibition effects compared to its predecessor [9]. Acalabrutinib has the property of selectively inhibiting BTK without targeting antibody-dependent cellular phagocytosis (ADCP) and antibody-dependent cellular cytotoxicity (ADCC) mechanisms, which enables its use as monotherapy and in combination therapy with obinutuzumab through complementary mechanisms of action [10].

The ELEVATE-TN and ASCEND phase III trials have shown superior efficacy and a favourable safety profile compared to standard chemo-immunotherapy regimens in patients with treatment-naive and previously treated CLL, respectively [11, 12]. In the ELEVATE-TN trial, acalabrutinib with or without obinutuzumab significantly reduced the risk of disease progression or death by 90% and by 81% respectively, compared to chlorambucil plus obinutuzumab over a 47-month follow up period [13]. The estimated 48-month progression-free survival (PFS) rates after 4 years of follow-up were 87.0% for acalabrutinib-obinutuzumab, 77.9% for acalabrutinib alone, and 25.1% for the control group [6]. In the ASCEND trial, acalabrutinib significantly improved PFS compared to idelalisib plus rituximab (IdR) or bendamustine plus rituximab (BR) in relapsed/refractory (R/R) CLL, with 42-month PFS rates of 62% for acalabrutinib versus 19% for IdR/BR [14]. More recently, the ELEVATE R/R non-inferiority study compared acalabrutinib with ibrutinib with centrally confirmed del(17p) or del(11q) patients previously treated for CLL. Acalabrutinib demonstrated non-inferior PFS compared to ibrutinib in this study with an improved safety profile and fewer cardiovascular adverse events (AE) [15, 16].

To date, only a limited number of real-world (rw) studies have assessed acalabrutinib in CLL, primarily through extraction of data from electronic health record in U.S databases, with several limitations [17–19]. These analyses have demonstrated lower discontinuation rates for acalabrutinib compared to ibrutinib at 12 months that need to be consolidated with larger cohorts and longer follow-up [17, 19]. These initial findings highlight the necessity of complementing clinical trial data with real-world evidence (RWE) studies, particularly in populations that may have been excluded from clinical trials.

NAOS study (National Acalabrutinib Observational Study) is a large-scale RWE initiative [20] which objectives are to provide valuable evidence into the use of acalabrutinib in CLL patients in the real world by collecting data on patients’ characteristics, treatment patterns, and outcomes in hospital medical charts.

## Materials and methods

### Study design

NAOS is a retrospective, non-interventional, longitudinal, multicentre study conducted in 59 sites in France. It included patients with CLL, who initiated treatment with acalabrutinib, with or without obinutuzumab, in first line or beyond, in 2021 and 2022, within standard medical practice. The study is based on secondary data collection from hospital patients’ charts.

The study enrolled CLL adult patients (≥ 18 years) who initiated acalabrutinib at the physician’s discretion whether they were still under or discontinued the treatment at the time of their enrolment. At the time of the study, acalabrutinib use in France was restricted to specific patient populations. In November 2020, acalabrutinib received EMA approval for a broad indication, including use as monotherapy or in combination with obinutuzumab in first line (L1), and as monotherapy for second line (L2) and beyond (L2+). Reimbursement in France for these indications only began in March 2023. For patients initiating acalabrutinib treatment in France during 2021 and 2022 (up to June 2023), which includes the two cohorts of patients in NAOS, access was primarily through the French early access program. This program placed certain conditions on acalabrutinib use. For L1 patients without del(17p) and/or *TP53* mutation, acalabrutinib with or without obinutuzumab was limited to patients ineligible for fludarabine-based regimens. For L1 patients with del(17p) and/or *TP53* mutation, acalabrutinib monotherapy was only reimbursed for patients intolerant or ineligible for ibrutinib. For L2+, acalabrutinib was reimbursed as monotherapy in patients who had received as least one prior therapy for CLL.

Participating sites were requested to use standardized requests on appropriate internal medical software or pharmacy or hospital databases to screen and invite all eligible patients to participate in the study. Patients were informed of the study and did not oppose to their data collection before being enrolled, with adapted measures regarding deceased patients in accordance with local regulations.

Two successive enrolment periods were scheduled, in 2022 for patients who had initiated treatment in 2021 (set #1), and in 2023 for those who had initiated in 2022 (set #2). Follow-up was planned for 3 years from the start of treatment (baseline) or until study withdrawal (death, lost to follow-up, patient’s decision, physician’s choice, other reason), whichever occurred first. An update of patients’ data was recorded once a year and comprised all assessments performed in routine clinical practice during the previous year.

### Study objectives and endpoints

The primary objective was to estimate the time to discontinuation (TTD) of acalabrutinib treatment in real-life practice, and reasons for discontinuation, overall and according to treatment line. TTD was defined as the day when it was stopped for whatever reason, or death. Discontinuation was defined as any interruption of 28 consecutive days or more including death. Any restart of acalabrutinib after an interruption > 28 days was considered as a subsequent therapy. The discontinuation rate (patients who stopped using acalabrutinib) was assessed at 12 months (M12).

Secondary endpoints included rw efficacy with the assessment of rw progression free survival (rwPFS), defined as the time from the start of acalabrutinib to the date of investigator determined disease progression or death, and rw overall survival (rwOS), defined as the time from the start of acalabrutinib to the date of death, with assessment of patients’ event-free rates at 12 months, as well as description of rw patients’ characteristics and treatment use (dose reductions and increases, dose interruptions (≤ 28 days) and reasons).

Clinical safety assessments included all grade AEs leading to treatment changes (dose changes, temporary interruption, and discontinuation). AEs that did not result in treatment changes were not collected in this secondary data use study which was in line with European Good Pharmacovigilance Practice.

### Clinical assessment and data collection

The data were extracted from hospital patient charts issued from routine clinical practice. Patients were treated with acalabrutinib at the physician’s discretion in routine clinical practice. Follow-up visits occurred according to routine clinical practice. Retrospective data collection was performed once a year. At the time of the analysis, data were collected from routine medical records since the treatment start up to the end of 2022.

At the treatment start, patient demographic data, Oncology Group Performance Status (ECOG-PS), comorbidities and biological characteristics were described. CLL diagnosis and characteristics including time since diagnosis were collected. Cytogenetic status included chromosome 17p deletion (del(17p)), chromosome 11q deletion (del(11q)), complex karyotype (> 3 abnormalities), genomic status unmutated (u) or mutated (m) immunoglobulin heavy chain variable region genes (*IGHV*) and mutated *TP53* (m*TP53*), when available. Also, any previous treatments, concomitant medications, as well as acalabrutinib start date and posology, were recorded.

During follow-up visits, investigators recorded disease assessments, disease progression, and new comorbidities as per the normal clinical practice for the site. Subsequent therapies after acalabrutinib were recorded up to patient death or end of follow up, when applicable. Acalabrutinib treatment changes and reasons for dose change(s), including dose reduction and dose increase, interruption(s) and discontinuation were recorded. AE leading to changes were collected and coded using the Medical Dictionary for Regulatory Activities (MedDRA) version 25.0 terminology at a Preferred Term (PT) and System Organ Class (SOC) levels.

This study was conducted in accordance with the ethical principles of the Declaration of Helsinki, the principles of the International Society for Pharmacoepidemiology guidelines for Good Pharmacoepidemiology Practice, and in compliance with the General Data Protection Regulation (GDPR). The protocol was submitted to the French Health Data Hub (N° F20220503111322). No additional ethical approval was required for this study as per local regulation since the study is based on secondary data issued from medical charts. The study is recorded on clinicaltrial.gov (NTC number: NCT05437250).

#### Statistical analysis

The objective of this intermediate analysis is to describe the baseline characteristics of the overall study population (sets #1 and #2), and to assess the use, efficacy and safety of acalabrutinib at 12 months for patients of set #1 who started acalabrutinib-based treatments in 2021.

All endpoints were analysed in the eligible population. The safety population included all patients of set #1 who received at least one dose of acalabrutinib. Baseline patient characteristics, treatment use and safety data are presented with descriptive statistics. Qualitative data are presented as numbers with the corresponding percentages while quantitative data are presented as means with standard deviation (SD) and/or median with interquartile range (IQR). The numbers of patients with missing data are indicated. Missing data were not replaced.

Time-to-event analyses (TTD, rwPFS, rwOS) were estimated using the Kaplan-Meier method, the 95% CI were estimated using the Greenwood formulae. Survival differences were compared in subgroups using the log rank test. Patients who were event-free at the end of the study were censored at the last date of disease assessment. Analyses were assessed for the total population and for subgroups: line of treatment (LOT) (L1 and L2+), age (< 75 years and ≥ 75 years), del(17p) and/or *TP53* genetic status (yes and no), *IGHV* status (unmutated (u) and mutated (m)) and cardiovascular comorbidities (yes and no). Statistical analyses were conducted using SAS version 9.4 (SAS Institute Inc., Cary, NC, USA). Annual analyses were planned.

## Results

### Baseline patients’ characteristics

In total, 501 patients were enrolled in the study between November 2022 and January 2023 by the participating sites. Sixteen patients were excluded from the analyses of which 1 had not been administered acalabrutinib, 13 did not meet eligibility criteria and 2 had no inclusion visit completed. At the time of the present analysis, 485 (96.4%) patients had their baseline data retrospectively completed. Of them, 188 (38.8%) patients initiated acalabrutinib in 2021 (set #1) and 297 (61.2%) in 2022 (set #2). The median follow-up (from treatment start to data collection) was 12.0 months (IQR, 7.7–16.4), 17.5 months (IQR, 13.9–20.2) and 8.5 months (IQR, 5.6–11.7) for set#1 and set#2, respectively.

Patient demographics and disease characteristics at baseline are summarized in Table 1 for the complete cohort. Of them, 285 patients initiated acalabrutinib at L1 (58.8%) and 200 (41.2%) at L2+, including 109 patients at L2 and 91 at L3+. The median age at inclusion was 73 and 77 years in the L1 and L2 + subgroups respectively. Half of the patients (50.9%) were 75-years or above with a median age of 80 years (IQR, 77–85) for this specific subgroup. Among the 383 patients with ECOG data, 16.7% had an ECOG-PS ≥ 2.

**Table 1:** Patient’s baseline characteristics of the overall NAOS population

	**L1** **(** ***N*** **=285)**		**L2+** **(** ***N*** **=200)**		**Total** **(** ***N*** **=485)**	
Age at initiation (years)						
median (IQR)	73	(69–79)	77	(70–3)	75	(70–81)
< 65, *n* (%)	40	(14.2)	18	(9.0)	58	(12.0)
≥ 75, *n* (%)	130	(45.6)	117	(58.5)	247	(50.9)
Male, *n* (%)	186	(65.3)	129	(64.5)	315	(64.9)
ECOG PS, *n* (%)*	***N*** **=**224		***N*** **=**159		***N*** **=**383	
0	106	(47.3)	62	(39.0)	168	(43.9)
1	89	(39.7)	62	(39.0)	151	(39.4)
≥ 2	29	(12.9)	35	(22.0)	64	(16.7)
Prior cardiovascular comorbidities*	***N*** **=**278		***N*** **=**196		***N*** **=**474	
Yes (at least one), *n* (%)	150	(54.0)	111	(56.6)	261	(55.1)
Myocardial infarction, *n* (%)	14	(5.0)	14	(7.1)	28	(5.9)
Congestive heart failure, *n* (%)	10	(3.6)	10	(5.1)	20	(4.2)
Peripherical vascular disease, n (%)	28	(10.1)	11	(5.6)	39	(8.2)
Hypertension, *n* (%)	121	(43.5)	90	(45.9)	211	(44.5)
Cardiac rhythm disorders, *n* (%)	26	(9.4)	33	(16.8)	59	(12.4)
* Including Atrial fibrillation*	*18*	*(6.3)*	*23*	*(11.5)*	*41*	*(8.5)*
* Atrial flutter*	*3*	*(1.1)*	*2*	*(1.0)*	*5*	*(1.0)*
Treated with antithrombotic agent *n* (%)	82	(28.8)	68	(34.0)	150	(30.9)
Creatinine clearance (mL/min)*	***N*** **=**209		***N*** **=**132		***N*** **=**341	
Median (IQR)	72.7	(57–83)	63.7	(51–78)	70.0	(54–82)
< 60 mL/min	59	(28.2)	55	(41.7)	114	(33.4)
Time from diagnosis of CLL (years)						
median (IQR)	3.0	(0.9–6.4)	9.1	(5.3–13.2)	5.1	(1.9–9.9)
BINET assessment, *n* (%)	***N*** =220		***N*** =135		***N*** =355	
Stade A	27	(12.3)	20	(14.8)	47	(13.2)
Stade B	113	(51.4)	60	(44.4)	173	(48.7)
Stade C	80	(36.4)	55	(40.7)	135	(38.0)
High-risk cytogenetic features*						
Del(17p) *	***N*** =207		***N*** =135		***N*** =342	
Yes, *n* (%)	36	(17.5)	36	(26.9)	72	(21.2)
Mutated TP53*	***N*** =225		***N*** =137		N=362	
Yes, *n* (%)	35	(15.8)	34	(25.6)	69	(19.4)
Del(17p) and/or *TP53, n* (%)	***N*** =262		***N*** =150		***N*** =412	
Yes, *n* (%)	52	(19.8)	55	(33.3)	107	(25.1)
Del(17p) and *TP53, n* (%)	***N*** =262		***N*** =150		***N*** =412	
Yes, *n* (%)	19	(7.3)	15	(9.1)	34	(8.0)
Unmutated IGHV*	***N*** =189		***N*** =87		***N*** =276	
Yes, n (%)	120	(65.2)	62	(72.1)	182	(67.4)
Del (11q)	***N*** =177		***N*** =115		***N*** =292	
Yes, *n* (%)	48	(27.1)	46	(40.4)	94	(32.3)
Complex karyotype*,**	***N*** =150		***N*** =84		***N*** =234	
Yes, *n* (%)	43	(28.7)	36	(43.4)	79	(33.9)
Prior therapies, *n* (%)			***N*** =200			
Prior ibrutinib, *n* (%)	-		71	(35.5)	-	
Prior venetoclax, *n* (%)	-		31	(15.5)	-	
Prior CIT, *n* (%)	-		135	(67.5)	-	
Type of treatment received, *n* (%)						
Acalabrutinib in monotherapy	219	(76.8)	186	(93.0)	405	(83.5)
Acalabrutinib with Obinutuzumab	63	(22.1)	11	(5.5)	74	(15.3)
Acalabrutinib with other	3	(1.1)	3	(1.5)	6	(1.2)

*Based on observed data (unknown/unavailable data are not included in observed data); **> 3 abnormalities

More than half of patients (55.1%) had at least one cardiovascular comorbidity, 44.5% of whom had hypertension, 12.4% cardiac rhythm disorders (including 8.5% of atrial fibrillation). Cardiac rhythm disorder was present in 9.4% of L1 patients and 16.8% of ≥ L2 patients. In L1 patients, rates were 45.7% (< 75 years) and 63.8% (≥ 75 years), while in L2 + patients, they were 45.1% (< 75 years) and 64.9% (≥ 75 years). Regarding genetic characteristics, del(17p) was present in 21.2%, *TP53*m in 19.4%, del17p/TP53m in 25.1% (19.8% in L1 and 33.3% in L2 + subgroups), del(11q) in 32.3% and u*IGHV* in 67.4% of patients (65.2% in L1 and 72.1% in L2 + subgroups), respectively.

### Treatment initiation

Acalabrutinib was initiated as monotherapy in 76.8% and 93.0% of L1 and L2 + patients, respectively and associated with obinutuzumab in 22.1% and 5.5% in L1 and L2 + patients, respectively. In L1, obinutuzumab was more frequently associated with acalabrutinib in younger patients (27.7% in < 75 years and 15.4% in ≥ 75 years patients). Its use was rare in L2 + patients whatever the age class (8.4% in < 75 years and 3.4% in ≥ 75 years subgroups). Most patients (93.5%) initiated acalabrutinib treatment at the recommended dosage of 100 mg twice daily; however, a subset of patients in L1 and L2+, 4.6% (*n* = 13) and 9.0% (*n* = 18) respectively, started the therapy at a reduced dosage of 100 mg once daily.

In addition, many patients have been on concomitant treatment for their baseline diseases including 36.1% for hypertension (38.6% in L1 and 42.5% in L2+), 9.1% for atrial fibrillation (7.3% in L1 and 11.5% in L2+) and 30.9% received antithrombotic agents (28.8% in L1 and 34.0% in L2+). Overall, patients ≥ 75 years received more concomitant treatments than younger patients including antithrombotic agents used in 16.8% and 17.8% of L1 and L2 + patients < 75 years, and in 32.3% and 35.9% of L1 and L2 + patients ≥ 75 years, respectively.

Baseline characteristics for set #1 and set #2 were similar in terms of age, *sex ratio*, ECOG, time since diagnosis, genetic abnormalities and Binet classification. Of note, the set #1 of 188 patients initiated in 2021 comprised 119 patients in L1 (63%) and 69 in L2+ (37%) and the set#2 of 297 patients initiated in 2022 comprised 166 patients in L1 (56%) and 131 in L2+ (44%). In addition, 45.2% and 60.1% of L1 set#1 and L1 set#2 had cardiovascular comorbidities, respectively. Conversely, 64.7% and 52.3% of the L2 + set #1 and L2 + set#2 patients had cardiovascular comorbidities, respectively. Obinutuzumab was associated with acalabrutinib in 25.5% and 19.9% of L1 set#1 and set#2 respectively and in 4.3% and 6.1% of L2 + set#1 and set#2, respectively.

### Administration of treatment and time to discontinuation of treatment (TTD)

Time to discontinuation was assessed in patients of set #1. Of these 188 patients, 59 (31.4%) discontinued treatment for any reason or death. The reasons for discontinuation were AE (28 patients, 47.5%), physician’s choice (4 patients, 6.8%), disease progression (3 patients, 5.1%) and other reasons (medical procedure) (2 patients, 3.3%). In 22 patients (37.3%), treatment discontinuation was associated with death, mainly in patients ≥ 75 years (17/97, 17,5%) compared to patients < 75 years (5/91, 5.5%). Deaths in patients ≥ 75 years occurred in 7/52 (13.5%) in L1 and in 10/45 (22.2%) in L2+.

The discontinuation rate at M12 was 22.7% (95% CI, 16.9–29.0). The discontinuation rate was not statistically associated to line of treatment (LOT) (L1: 21.5%, (95% CI, 14.5–29.3) vs. L2: 24.7%, (95% CI, 15.2–35.4), *p* = 0.27). When the L2 + subgroup is separately assessed in L2 (*n* = 109) and L3+ (*n* = 91), the discontinuation rate at M12 was 19.4%, (95% CI, 8.4–33.8) in the L2 patients and 30.4% (95% CI, 15.7–46.6%) in the later lines, respectively. The discontinuation rate was not associated with cardiovascular (CV) comorbidities (No CV comorbidity: 17.5%, (95% CI, 10.3–26.3) vs. CV comorbidity: 27.4%, (95% CI, 18.8–36.7), *p* = 0.11) nor with the age class (< 75 years: 17.8%, (95% CI, 10.7–26.4) vs. ≥ 75 years: 27.3%, (95% CI, 18.7–36.5), *p* = 0.11).

**Fig. 1:**
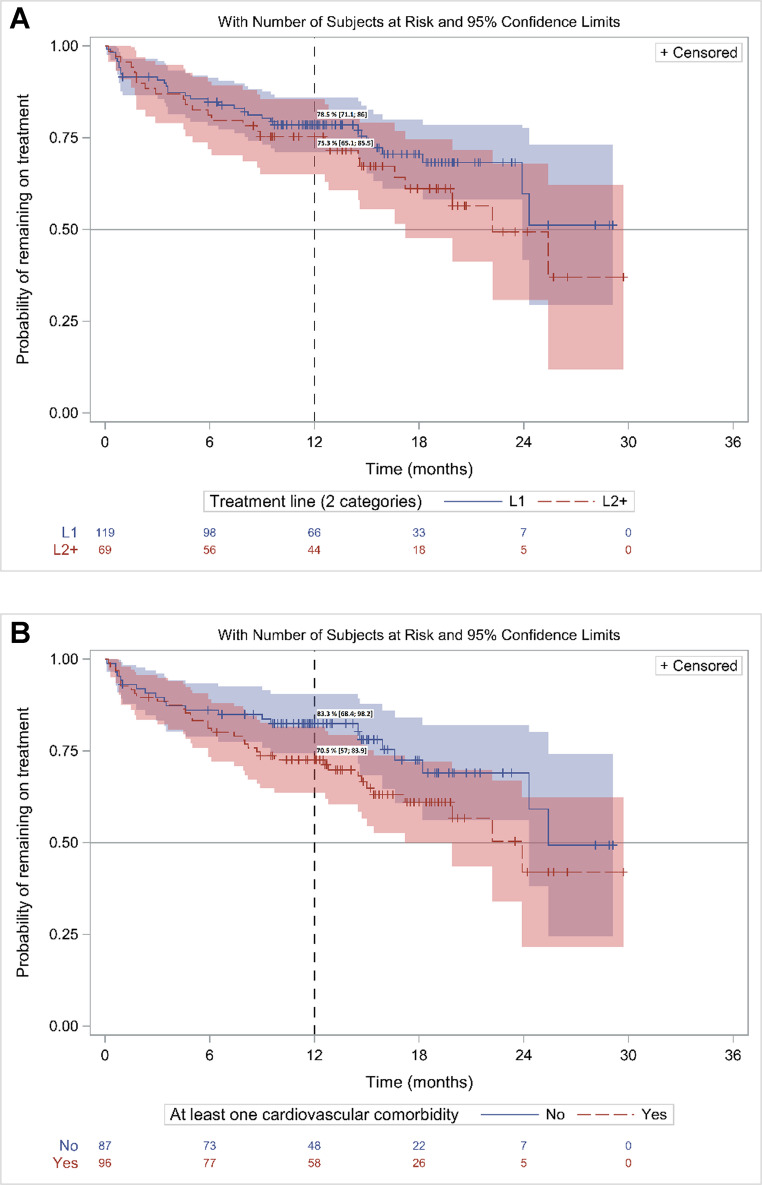
TTD in patients initiating acalabrutinib in 2021 (Kaplan Meier): A/in L1 and L2 + patients; B/in all patients (L1&L2+) with and without CV comorbidities

Thirteen patients (6.9%) had their dose of acalabrutinib reduced during treatment, at a median of approximately 6.5 months (IQR, 2.7–12.3) after the start of treatment, due to AE or other physician decision. Eight patients (4.2%) had their dose increased at a median of approximately 1.8 months (IQR, 1.0–2.8) after the start of treatment, due to physicians’ decision (Fig. 1).

#### Efficacy

##### Rw progression free survival (rwPFS)

**Fig. 2:**
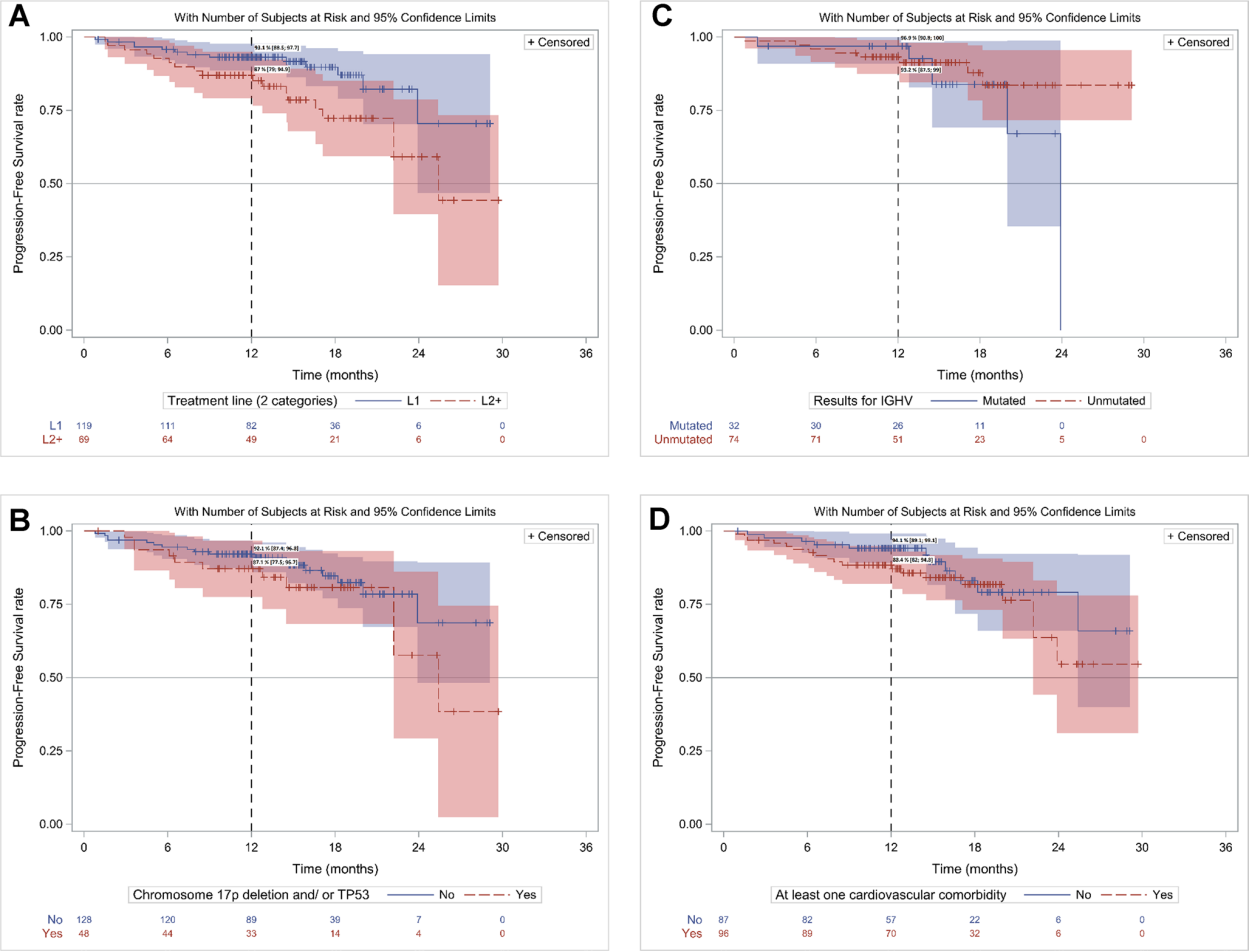
rwPFS in patients initiating acalabrutinib in 2021 (Kaplan Meier): A/ in L1 and L2+ patients; B/ in all patients (L1&L2+) with 17p deletion and/ or TP53 mutation and without; C/ in IGHVm and in uIGHV all patients (L1&L2+); D/ in all patients (L1&L2+) w and wo CV comorbidities

Among set #1, 157 patients were progression-free at the time of the analysis (106/119 in L1 and 51/69 in L2+). The rwPFS rate at M12 was 90.8% (95% CI, 86.6–95.0) for the overall population, 93.1% (95% CI, 88.5–97.7) in L1 vs. 87% (95% CI, 79.0-94.9) in L2 + patients (*p* = 0.02) (Fig. 2A). The median rwPFS was not reached (NR).

The rwPFS was similar in subgroup analyses including patients with high-risk cytogenetic or genomic characteristics. Patients with and without del17p/*TP53*m had similar PFS (*p* = 0.21) with M12 rwPFS rates of 92.1% (95% CI,87.4–96.8) vs. 87.1 (95% CI, 77.5–96.7) respectively (Fig. 2B). In the same way, the rwPFS was similar in *IGHV*m and u*IGHV* patients (*p* = 0.34) with M12 rwPFS rates of 96.9% (95% CI, 90.8–100) vs. 93.2% (87.5–99.0), respectively (Fig. 2 C). The rwPFS was similar in patients with and without CV comorbidities (*p* = 0.26) (M12 rwPFS rate: 94.1% (95% CI,,89.1–99.1) vs. 88.4 (95% CI, 82.0-94.8)), respectively (Fig. 2D).

#### Overall survival (OS)

**Fig. 3:**
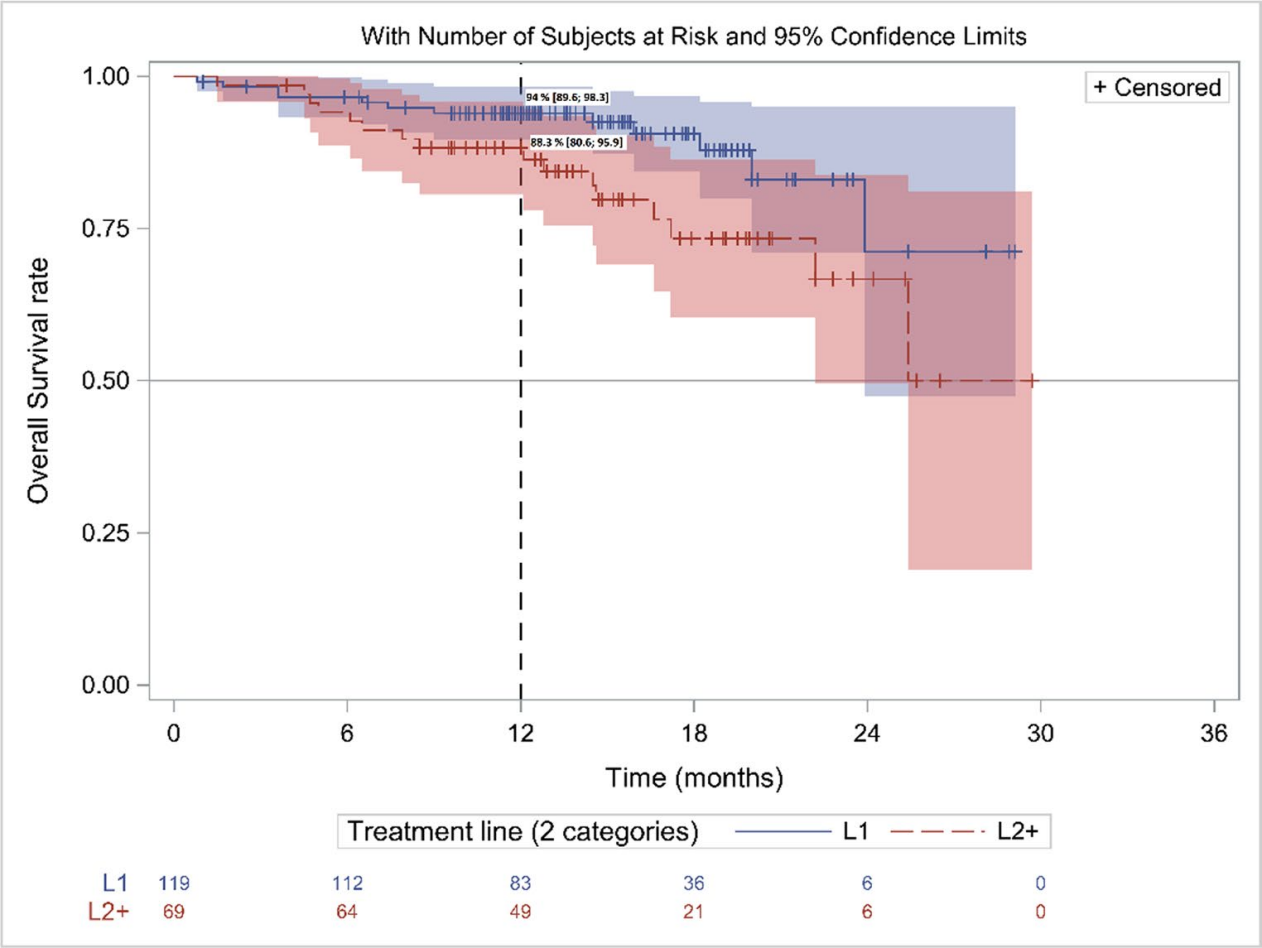
rwOS in patients initiating acalabrutinib in 2021 (Kaplan Meier): A/ in L1 & L2+ patients

During the analysis study period, 28 patients died (14.9%), 12 in L1 patients (10.9%) and 16 in L2+ (23.2%) including 7 in L2 (19.4%) and 9 (27.3%) in L3+. The median OS was NR. The rwOS rate at M12 was 91.8% (95% CI, 87.9–95.8) for the overall population, 94.0% (95% CI, 89.6–98.3) in L1 vs. 88.3% (95% CI, 80.6–95.9) in L2 + patients (*p* = 0.04) (Fig. 3). of the 28 patients who died, 32,1% of patients were < 75 years and 67,9% ≥ 75 years, mostly in L2 + patients.

##### Safety

AEs leading to treatment changes (dose reduction, treatment interruption or discontinuation) were identified in 40 of 188 patients of set #1 (21.3%) (Table 2). This includes 8 patients (4.3%) with AE leading to dose changes, 13 patients with AE leading to treatment interruption (6.9%) and 24 patients (12.8%) with AE leading to treatment discontinuation as reported by investigators. Of these 40 patients, 18 patients experienced grade 3/4 AE leading to treatment changes (Table 3).

Haematological disorders were the most frequent AE, reported in 9 patients (4.8%) including 4 patients with grade 3/4 AE (three patients with neutropenia and one with unspecified haematotoxicity) and treatment discontinuation in 3 patients. In addition, 6 patients (3.2%) experienced vascular AE including 5 cases of grade 1/2 haemorrhage; most (5/6) resulted in acalabrutinib discontinuation. Among these 6 patients with vascular AE, 3 were receiving anticoagulant therapy at baseline. Overall, 3 out of the 52 patients exposed to antithrombotic agents (5.8%) and 3 out of the 136 patients not exposed (2.2%), experienced vascular adverse events that led to treatment modifications.

Infection-related AE led to treatment changes in 7 patients (3.7%) including 4 patients experiencing grade 3/4 AE (two patients with coronavirus infection, one with brain abscess, one with septic shock); 4 of them led to treatment discontinuation. Six patients reported headaches none of which were grade 3/4, but 4 led to treatment discontinuation.

Cardiac disorders AEs leading to treatment changes occurred in 4 patients (2.1%), including one patient each with angina pectoris, atrial fibrillation, myocardial infarction, and tachyarrhythmia; half of these cases resulted in treatment discontinuation. No cases of hypertension leading to treatment change were reported. The myocardial infarction and angina pectoris cases were classified as grade 3/4 AEs and resulted in treatment discontinuation but were non-fatal. Among those 4 patients, 3 had hypertension at baseline, and none had a history of atrial fibrillation. Of the 188 set#1 patients, 87 (46.2%) had either hypertension or atrial fibrillation at baseline. Cardiac disorder AEs occurred in 3 of them, and in 1 patient without these conditions.

A single non-treatment-related AE leading to death was reported and was attributed to general physical health deterioration.

**Table 2:** Description of AEs (all grades) leading to treatment changes by SOC classes and Preferred Terms (PT) per SOC in patients of set #1 (*n*=188)

	AE leading to							
	any change*		**Dose reduction**		**Interruption**		**Discontinuation**	
	***n***	**%**	***n***	**%**	***n***	**%**	***n***	**%**
Patients with AE of any SOC	40	21.3	8	4.3	13	6.9	24	12.8
Blood and lymphatic system disorders	9	4.8	2	1.1	6	3.2	3	1.6
Neutropenia	3	1.6	-	-	3	1.6	1	0.5
Anaemia	2	1.1	-	-	2	1.1	1	0.5
Thrombocytopenia	2	1.1	1	0.5	-	-	1	0.5
Haematotoxicity	1	0.5	1	0.5	-	-	-	-
Splenic haematoma	1	0.5	-	-	1	0.5	-	-
Infections and infestations	7	3.7	1	0.5	3	1.6	4	2.1
Coronavirus infection	3	1.6	1	0.5	2	1.1	1	0.5
Actinomycosis	1	0.5	-	-	.	.	1	0.5
Beta haemolytic streptococcal infection	1	0.5	-	-	1	0.5	-	-
Brain abscess	1	0.5	-	-	-	-	1	0.5
Citrobacter infection	1	0.5	-	-	1	0.5	-	-
Septic shock	1	0.5	-	-	-	-	1	0.5
Nervous system disorders	6	3.2	1	0.5	3	1.6	4	2.1
Headache	6	3.2	1	0.5	3	1.6	4	2.1
Vascular disorders	6	3.2	1	0.5	1	0.5	5	2.7
Haemorrhage	5	2.7	1	0.5	1	0.5	4	2.1
Haematoma	1	0.5	-	-	-	-	1	0.5
Cardiac disorders	4	2.1	1	0.5	1	0.5	2	1.1
Angina pectoris	1	0.5	-	-	-	-	1	0.5
Atrial fibrillation	1	0.5	1	0.5	-	-	-	-
Myocardial infarction	1	0.5	-	-	-	-	1	0.5
Tachyarrhythmia	1	0.5	-	-	1	0.5	-	-
General disorders and administration site conditions	4	2.1	.	.	.	.	4	2.1
Asthenia	2	1.1	-	-	-	-	2	1.1
General physical health deterioration	2	1.1	-	-	-	-	2	1.1
Skin and subcutaneous tissue disorders	3	1.6	.	.	2	1.1	1	0.5
Ecchymosis	1	0.5	-	-	-	-	1	0.5
Eczema	1	0.5	-	-	1	0.5	-	-
Rash maculo-papular	1	0.5	-	-	1	0.5	-	-
Gastrointestinal disorders	2	1.1	.	.	.	.	2	1.1
Diarrhoea	1	0.5	-	-	.	.	1	0.5
Gastrointestinal disorder	1	0.5	-	-	.	.	1	0.5
Injury, poisoning and procedural complications	2	1.1	.	.	2	1.1	.	.
Eye contusion	1	0.5	-	-	1	0.5	-	-
Post procedural haematoma	1	0.5	-	-	1	0.5	-	-
Metabolism and nutrition disorders	2	1.1	2	1.1	.	.	.	.
Hyponatraemia	2	1.1	2	1.1	-	-	-	-
Eye disorders	1	0.5	.	.	1	0.5	1	0.5
Diplopia	1	0.5	-	-	1	0.5	1	0.5
Investigations	1	0.5	.	.	.	.	1	0.5
Electrocardiogram abnormal	1	0.5	-	-	-	-	1	0.5
Musculoskeletal and connective tissue disorders	1	0.5	.	.	.	.	1	0.5
Back pain	1	0.5	-	-	-	-	1	0.5
Neoplasms benign, malignant and unspecified	1	0.5	.	.	.	.	1	0.5
Lung adenocarcinoma	1	0.5	-	-	-	-	1	0.5
Respiratory, thoracic and mediastinal disorders	1	0.5	.	.	.	.	1	0.5
Acute respiratory failure	1	0.5	-	-	-	-	1	0.5
Not specified**	2	1.1	1	0.5	1	0.5	1	0.5

*Any change includes dose change or interruption or discontinuation - one AE can lead to different changes

**No more information at the time of the interim analysis – *SOC*: System Organ Class according to MedDRA V25.0

**Table 3:** Description of AEs (grade 3/4) leading to treatment changes by SOC classes and Preferred Terms (PT) per SOC in patients of set #1 (*n*=188)

	**Grade 3/4 AE leading to**							
	Any change*		**Dose reduction**		**Interruption**		**Discontinuation**	
	***n***	**%**	***n***	**%**	***n***	**%**	***n***	**%**
Patients with AE of any SOC	18	9.6	3	1.6	7	3.7	11	5.9
Blood and lymphatic system disorders	4	2.1	1	0.5	3	1.6	1	0.5
Neutropenia	3	1.6	-	-	3	1.6	1	0.5
Haematotoxicity	1	0.5	1	0.5	-	-	-	-
Infections and infestations	4	2.1	-	-	1	0.5	3	1.6
Coronavirus infection	2	1.1	-	-	1	0.5	1	0.5
Brain abscess	1	0.5	-	-	-	-	1	0.5
Septic shock	1	0.5	-	-	-	-	1	0.5
Cardiac disorders	2	1.1	-	-	-	-	2	1.1
Angina pectoris	1	0.5	-	-	-	-	1	0.5
Myocardial infarction	1	0.5	-	-	-	-	1	0.5
Eye disorders	1	0.5	-	-	1	0.5	1	0.5
Diplopia	1	0.5	-	-	1	0.5	1	0.5
Gastrointestinal disorders	1	0.5	-	-	-	-	1	0.5
Diarrhoea	1	0.5	-	-	-	-	1	0.5
General disorders and administration site conditions	1	0.5	-	-	-	-	1	0.5
General physical health deterioration	1	0.5	-	-	-	-	1	0.5
Injury, poisoning and procedural complications	1	0.5	-	-	1	***0.5***	***-***	***-***
Post procedural haematoma	1	0.5	-	-	1	0.5	-	-
Metabolism and nutrition disorders	1	0.5	1	0.5	-	***-***	-	-
Hyponatraemia	1	0.5	1	0.5	-	-	-	-
Neoplasms benign, malignant and unspecified	1	0.5	-	-	-	-	1	0.5
Lung adenocarcinoma	1	0.5	-	-	-	-	1	0.5
Respiratory, thoracic and mediastinal disorders	1	0.5	-	-	-	-	1	0.5
Acute respiratory failure	1	0.5	-	-	-	-	1	0.5
Not specified**	2	1.1	1	0.5	1	0.5	1	0.5

*Any change includes change or interruption or discontinuation - one AE can lead to different changes

**No more information at the time of the interim analysis - SOC: System Organ Class according to MedDRA V25.0

## Discussion

NAOS study represents an important contribution in RWE for the use of acalabrutinib in CLL which was the first second-generation BTKis approved in Europe in November 2020. Although previous analyses have been reported from US claimed databases [17–19], NAOS is, to our knowledge, the first comprehensive rw cohort study of its size, including both treatment-naive and R/R patients, in Europe [20]. With nearly 500 patients enrolled and followed by clinicians, NAOS is now yielding its initial results.

As expected in rw settings, patients receiving acalabrutinib had a broader and less standardized profile compared to those in clinical trials. NAOS study reveals an older population, with a median age of 75 years at acalabrutinib initiation (73 for L1 and 77 for L2 + patients) that differs from clinical trials where CLL treatment-naïve patients had a median age of 70 years [11], and where R/R patients were 66–68 years old in ELEVATE RR and ASCEND studies [12, 15]. Notably, patients aged 75 and older constituted half of the treated population (46% in L1 and 59% in L2+) in NAOS, compared to 28% of treatment-naïve and 16–22% of R/R patients in clinical trials [11, 12, 15]. Additionally, NAOS rw patients more frequently presented with impaired ECOG status (≥ 2) (12.9-22% in R/R patients) versus clinical trials (7% in first-line, 8–12% in L2+) [11, 12, 15], which has been shown as an unfavourable factor [21, 22]. Over half (55%) of patients had cardiac comorbidities or hypertension, 12% had a history of rhythm disorders, 6% had a history of myocardial infarction at treatment initiation and over 30% of patients received antithrombotic treatments. Patients with significant cardiovascular disorders or with concomitant warfarin or equivalent vitamin K antagonist treatment were excluded from clinical trials [11, 12, 15], and limited data are available for this population, despite their relevance given the known effects of first-generation BTK inhibitors.

Few rw cohorts had published initial results to date, apart from some preliminary data on small samples from early interim analyses [23, 24] and claimed database analyses. In the first rw studies, patients receiving acalabrutinib were 72 years old in first-line treatment [18, 19], and 70–72 years in predominantly R/R studies [17, 24]. In Europe, the retrospective EPIC study, issued from an early access program in the UK, included 54 first-line patients treated with acalabrutinib, with a median age of 74.5 years and an ECOG ≥ 2 (12% of patients), characteristics that are similar to NAOS [23]. For comparison, rw evidence studies previously performed with other BTK inhibitors (ibrutinib) showed consistent results with median ages ranging from 62 to 77 years [25]. Data on older populations with comorbidities and frail profiles are necessary as they reflect real-life practice [5].

The 12-month discontinuation rate for acalabrutinib treatment in NAOS study was 22.7% overall, with no significant difference between treatment-naïve (21.5%) and L2+ (24.7%) patients. These findings are consistent with other rw studies, despite some variations in definitions of treatment discontinuation (19% in EPIC [23], 22% in Roeker et al. [17], 19% in Yazdy et al. [24]). NAOS used a conservative approach, considering any interruption longer than 28 days as discontinuation and including deaths as events, which was not always the case in other studies. The most frequent reason for early discontinuation was AE, aligning with previously published studies [23, 24] and clinical trials [16]. Subgroup analyses showed no significant differences in discontinuation rates based on age, cardiovascular comorbidities, or treatment line, confirming good tolerability in high-risk subgroups. Moreover, this discontinuation rate is low in comparison with discontinuation rates of Ibrutinib in clinical trials and in real life studies [26] and it would be interesting to understand if it will favourably impact OS with further follow-up and in other studies.

The 12-month rwPFS estimated in NAOS (93.1% in L1 and 87% in L2+) were high and consistent with clinical trial results [11, 12, 15]. Notably, rwPFS did not differ significantly between patients with high-risk genomic factors (del(17p)/*TP53*m or u*IGHV*) and those without further supporting acalabrutinib effectiveness across various patient subgroups in rw settings. Although these 12-month results need to be confirmed with longer follow-up in upcoming analyses, they were consistent with Davids et al.’s pooled analysis that demonstrated high PFS and OS rates with acalabrutinib-based regimens in treatment-naive and R/R patients with higher-risk genomic features, including del(17p)/*TP53*m or u*IGHV* [24]. Rw results for these high-risk populations were not yet available from US databases or retrospective studies, and NAOS provides the first data in these patients outside of clinical trials ([17]– [18, 23]).

The pivotal clinical trials of acalabrutinib reported a more favourable AE profile compared to the ibrutinib experience [9]. The initial findings from the NAOS study, although retrospective and focused specifically on AEs leading to treatment changes, tend to support these conclusions. By focusing on AEs that result in dose adjustments, treatment interruptions, or discontinuations, the NAOS approach ensured a more comprehensive and robust record of clinically significant AEs in medical files, as these events were systematically associated with treatment modifications. These AEs may be considered particularly relevant, as they directly impact the continuation of treatment as initially prescribed.

Consistent with data from clinical trials and pooled analyses [16, 27–29], NAOS study observed few cardiac AE necessitating treatment modifications during the treatment period. Notably, no cases of hypertension led to treatment changes, and only one non-severe atrial fibrillation case was documented, although half of patients presented with cardiovascular comorbidities or hypertension at acalabrutinib initiation. These results corroborate the pooled analysis by Brown et al. [28], which demonstrated a comparatively low incidence of cardiac adverse reactions in 762 CLL patients treated with acalabrutinib monotherapy, an absence of hypertension-induced treatment discontinuations, and a reduced frequency of atrial fibrillation events relative to ibrutinib therapy.

More broadly, AE leading to treatment discontinuation were observed in 12.8% of patients, aligning with previous studies and rw data, such as the Roeker study, which reported a 12.7% discontinuation rate due to AEs, and a meta-analysis of five clinical trials indicating a 16.5% rate [27], with infections and neutropenia being the most common reasons for discontinuation. In NAOS study, infections, haematological and vascular disorders were also the primary reasons for discontinuation due to AEs, each accounting for 2.1% of patients. Of note, that this low rate of infections was reported despite 15.3% of patients receiving acalabrutinib plus obinutuzumab in NAOS study. Additionally, headaches contributed to four out of six discontinuation cases reported. Headaches are a common AE associated with acalabrutinib treatment [29] as previously observed. They were predominantly mild, with none classified as grade 3 or higher in NAOS study.

The study has limitations inherent to its retrospective, medical chart-based design. Relying on available patient records may result in incomplete data, as examinations and tests were not uniformly performed or documented in clinical practice across participating sites. Cytogenetic analysis, for instance, was not available for all treatment initiations. The AE occurrence might be incomplete for non-severe AE managed in outpatient settings but not reported in medical records. As a result, only AEs leading to treatment changes were captured in the study. These initial results encompassed half of the cohort, representing the first acalabrutinib-treated patients in France. The analysis of patients treated in 2021 could overrepresent those at advanced disease stages or older patients, particularly given the reimbursement restrictions at the time, and potentially biasing results towards less favourable outcomes. Finally, this first interim analysis had limited follow-up duration to assess long-term outcomes such as progression-free and overall survival in CLL and upcoming analyses will provide additional results on effectiveness.

## Conclusion

Overall, the NAOS study, with its large cohort of acalabrutinib-treated patients in Europe, provides relevant data on the real-world effectiveness and safety of acalabrutinib in CLL management, particularly in understudied populations. Encompassing nearly 500 patients across treatment-naive and R./R settings, the study demonstrates that acalabrutinib maintains its effectiveness in an older patient population with more comorbidities than typically seen in clinical trials. The safety profile data corroborates previous findings, showing a low incidence of cardiac adverse events including for patients with pre-existing cardiovascular conditions. As long-term follow-up data become available, NAOS will provide compelling real-world evidence on acalabrutinib in CLL management.

## Author contribution

AQ, SL, MSD, LB, ZM, OB, RG, AS, CD, PF, DRW, GD, KL, LY, VL were investigators in the study. AQ, SL, MSD, VL, and LY contributed to the protocol development, review and interpretation of analyses, and data exploitation. AQ, SL, MSD, LB, ZM, OB, RG, AS, CD, PF, DRW, GD, KL, LY, VL were involved in the writing review and approved the final version of the manuscript for publication.

## Data Availability

The data that support the findings of this study are not openly available due to reasons of sensitivity and are available from the corresponding author upon reasonable request.

## References

[1] Eichhorst B, Robak T, Montserrat E et al (2021) Chronic lymphocytic leukaemia: ESMO clinical practice guidelines for diagnosis, treatment and follow-up. Ann Oncol Off J Eur Soc Med Oncol 32(1):23–33 10.1016/j.annonc.2020.09.019 33091559

[2] Hallek M, Al-Sawaf O (2021) Chronic lymphocytic leukemia: 2022 update on diagnostic and therapeutic procedures. Am J Hematol 96(12):1679–1705 34625994 10.1002/ajh.26367

[3] Miranda-Filho A, Piñeros M, Ferlay J, Soerjomataram I, Monnereau A, Bray F (2018Jan) Epidemiological patterns of leukaemia in 184 countries: a population-based study. Lancet Haematol. 5(1):e14–e24. 10.1016/S2352-3026(17)30232-6 10.1016/S2352-3026(17)30232-6 29304322

[4] National Cancer Institute. Cancer Stat Facts: Leukemia—Chronic Lymphocytic Leukemia (CLL) [Internet]. Surveillance, Epidemiology, and End Results Program (SEER). [cited 2024 Oct 17]. Available from: https://seer.cancer.gov/statfacts/html/clyl.html

[5] Stauder R, Eichhorst B, Hamaker ME et al (2017) Management of chronic lymphocytic leukemia (CLL) in the elderly: a position paper from an international society of geriatric oncology (SIOG) task force. Ann Oncol 28(2):218–227 27803007 10.1093/annonc/mdw547

[6] Quinquenel A, Aurran-Schleinitz T, Clavert A et al (2020) Diagnosis and treatment of chronic lymphocytic leukemia: recommendations of the French CLL study group (FILO). HemaSphere 4(5):e473 33062946 10.1097/HS9.0000000000000473 PMC7523785

[7] Le Guyader-Peyrou S, Defossez G et al (2019) Estimations nationales de l’incidence et de la mortalité par cancer en France métropolitaine entre 1990 et 2018 - Volume 2– Hémopathies malignes: Étude à partir des registres des cancers du réseau Francim. Saint-Maurice (Fra): Santé publique France. ume-2-hemopathies-malignes. [cited 2024 Oct 17]

[8] Van der Straten L, Levin MD, Visser O et al (2021) Conditional relative survival among patients with chronic lymphocytic leukaemia: A population-based study in the Netherlands. EJHaem 3(1):180–183 35846209 10.1002/jha2.368 PMC9175753

[9] Tam C, Thompson PA (2024) BTK inhibitors in CLL: second-generation drugs and beyond. Blood Adv 8(9):2300–2309 38478390 10.1182/bloodadvances.2023012221 PMC11117011

[10] Woyach JA, Blachly JS, Rogers KA (2020) Acalabrutinib plus obinutuzumab in Treatment-Naïve and relapsed/refractory chronic lymphocytic leukemia. Cancer Discov 10(3):394–405 31915195 10.1158/2159-8290.CD-19-1130 PMC8176161

[11] Sharman EM, Jurczak W et al (2020) Acalabrutinib with or without obinutuzumab versus Chlorambucil and obinutuzumab for treatment-naive chronic lymphocytic leukaemia (ELEVATE-TN): a randomised, controlled, phase 3 trial. Lancet 18(10232):1278–1291 10.1016/S0140-6736(20)30262-2 PMC8151619 32305093

[12] Ghia P, Pluta A, Wach M et al (2020) ASCEND: phase III, randomized trial of acalabrutinib versus idelalisib plus rituximab or Bendamustine plus rituximab in relapsed or refractory chronic lymphocytic leukemia. J Clin Oncol 38(25):2849–2861 32459600 10.1200/JCO.19.03355

[13] Sharman JP, Egyed M, Jurczak W et al (2022) Efficacy and safety in a 4-year follow-up of the ELEVATE-TN study comparing acalabrutinib with or without obinutuzumab versus obinutuzumab plus Chlorambucil in treatment-naïve chronic lymphocytic leukemia. Leukemia 36(4):1171–1175 34974526 10.1038/s41375-021-01485-x PMC8979808

[14] Ghia P, Pluta A, Wach M et al (2022) Acalabrutinib versus investigator’s choice in relapsed/refractory chronic lymphocytic leukemia: final ASCEND trial results. Hemasphere 6(12):e801 36398134 10.1097/HS9.0000000000000801 PMC9666115

[15] Byrd JC, Hillmen P, Ghia P et al (2021) Acalabrutinib versus ibrutinib in previously treated chronic lymphocytic leukemia: results of the first randomized phase 3 trial. J Clin Oncol 39(31):3441–3452 34310172 10.1200/JCO.21.01210 PMC8547923

[16] Seymour JF, Byrd JC, Ghia P et al (2023) Detailed safety profile of acalabrutinib vs ibrutinib in previously treated chronic lymphocytic leukemia in the ELEVATE-RR trial. Blood 142(8):687–699 37390310 10.1182/blood.2022018818 PMC10644206

[17] Roeker LE, DerSarkissian M, Ryan K (2023) Real-world comparative effectiveness of acalabrutinib and ibrutinib in patients with chronic lymphocytic leukemia. Blood Adv 7(16):4291–4301 37163361 10.1182/bloodadvances.2023009739 PMC10424141

[18] Jacobs R, Lu X, Emond B (2024) Time to next treatment in patients with chronic lymphocytic leukemia initiating first-line ibrutinib or acalabrutinib. Future Oncol 20(1):39–53 37476983 10.2217/fon-2023-0436

[19] Hou JZ, Blanc S, Maglinte GA et al (2024) Real-world Bruton tyrosine kinase inhibitor treatment patterns and outcomes among patients with chronic lymphocytic leukemia or small lymphocytic lymphoma in US community oncology practices. 2024 EHA Congress, Presented at. Madrid, Spain

[20] Quinquenel A, Dilhuydy MS, Ysebaert L et al (2023) PB1925: NAOS study: first interim analysis of acalabrutinib use in patients with chronic lymphocytic leukemia in a real-life setting. Hemasphere 7(Suppl):e8766709

[21] Moldovianu AM, Stoia R, Vasilica M et al (2023) Real-World clinical outcomes and adverse events in patients with chronic lymphocytic leukemia treated with ibrutinib: A Single-Center retrospective study. Med (Kaunas) 59(2):324 10.3390/medicina59020324 PMC9959500 36837525

[22] Rigolin GM, Olimpieri PP, Summa V et al (2023) Outcomes in patients with chronic lymphocytic leukemia and TP53 aberration who received first-line ibrutinib: a nationwide registry study from the Italian medicines agency. Blood Cancer J 13(1):99 37380630 10.1038/s41408-023-00865-z PMC10307816

[23] Eyre T, Martinez-Calle N, Walewska R et al (2023) PB1930: EPIC: a non-interverional observational study of chronic lymphocytic leukemia patients treated with first-line acalabrutinib through the UK early access programme. Interim analysis up to 24 months. Hemasphere 7(Suppl):e980300b

[24] Yazdy MS, Mato AR, Roeker LE et al (2019) Toxicities and outcomes of Acalabrutinib-Treated patients with chronic lymphocytic leukemia: A retrospective analysis of real world patients. Blood 134(Supplement_1):4311. https://ashpublications.org/blood/article/134/Supplement_1/4311/424548/Toxicities-and-Outcomes-of-Acalabrutinib-Treated

[25] Lee P, Kistler KD, Douyon L et al (2023) Systematic Literature Review of Real-World Effectiveness Results Data for First-Line Ibrutinib in Chronic Lymphocytic Leukemia and Small Lymphocytic Lymphoma. Drugs Real World Outcomes 10(1):11–22 36534239 10.1007/s40801-022-00332-4 PMC9943824

[26] Ysebaert L, Quinquenel A, Bijou F et al (2020) Overall survival benefit of symptom monitoring in real-world patients with chronic lymphocytic leukaemia treated with ibrutinib: A FiLO group study. Eur J Cancer 135:170–172. 10.1016/j.ejca.2020.05.016 32585590 10.1016/j.ejca.2020.05.016

[27] Davids MS, Sharman JP, Ghia P et al (2024) Acalabrutinib-based regimens in frontline or relapsed/refractory higher-risk CLL: pooled analysis of 5 clinical trials. Blood Adv 8(13):3345–3359 38640349 10.1182/bloodadvances.2023011307 PMC11255369

[28] Brown JR, Byrd JC, Ghia P et al (2022) Cardiovascular adverse events in patients with chronic lymphocytic leukemia receiving acalabrutinib monotherapy: pooled analysis of 762 patients. Haematologica 107(6):1335–1346 34587719 10.3324/haematol.2021.278901 PMC9152976

[29] O’Brien SM, Brown JR, Byrd JC et al (2021) Monitoring and managing BTK inhibitor Treatment-Related adverse events in clinical practice. Front Oncol 11:720704 34858810 10.3389/fonc.2021.720704 PMC8630614

